# Laparoscopic gastrectomy with and without prophylactic drains in gastric cancer: a propensity score-matched analysis

**DOI:** 10.1186/s12957-019-1690-9

**Published:** 2019-08-16

**Authors:** Norihiro Shimoike, Shin Akagawa, Daisuke Yagi, Masazumi Sakaguchi, Yukinari Tokoro, Eiichiro Nakao, Takuya Tamura, Yusuke Fujii, Yuki Mochida, Yoshihisa Umemoto, Hidero Yoshimoto, Seiichiro Kanaya

**Affiliations:** Department of Surgery, Osaka Red Cross Hospital, 5-30, Fudegasaki, Tennouji-ku, Osaka, 543-8555 Japan

**Keywords:** Laparoscopic gastrectomy, Gastric cancer, Prophylactic drainage, Propensity score matching

## Abstract

**Background:**

The number of patients who are undergoing laparoscopic gastrectomy for treating gastric cancer is increasing. Although prophylactic drains have been widely employed following the procedure, there are few studies reporting the efficacy of prophylactic drainage. Therefore, this study assessed the efficacy of prophylactic drains following laparoscopic gastrectomy for gastric cancer.

**Methods:**

Data of patients who received laparoscopic gastrectomy for treating gastric cancer in our institution between April 2011 and March 2017 were reviewed, and the outcomes of patients with and without a prophylactic drainage were compared. Propensity score matching was used to minimize potential selection bias.

**Results:**

A total of 779 patients who underwent surgery for gastric cancer were reviewed; of these, 628 patients who received elective laparoscopic gastrectomy were included in this study. After propensity score matching, data of 145 pairs of patients were extracted. No significant differences were noted in the incidence of postoperative complications between the drain and no-drain groups (19.3% vs 11.0%, *P* = 0.071). The days after the surgery until the initiation of soft diet (6.3 ± 7.4 vs 4.9 ± 2.9 days, *P* = 0.036) and the length of postoperative hospital stay (15.7 ± 12.9 vs 13.0 ± 6.3 days, *P* = 0.023) were greater in the drain group than those in the no-drain group.

**Conclusions:**

This study suggests that routinely using prophylactic drainage following laparoscopic gastrectomy for treating gastric cancer is not obligatory.

## Background

Prophylactic abdominal drains have been widely employed following gastrointestinal surgeries. After gastric cancer surgery, prophylactic drains were used in 57.7 to 62.8% of the patients in observational studies [1, 2] and routinely used in a randomized controlled trial (RCT) [3]. The purpose of the prophylactic drain is to remove intra-abdominal fluid collections and to detect postoperative complications such as anastomotic leakage, intra-abdominal bleeding, and intra-abdominal abscess. Another potential function of prophylactic drains is in therapeutic drainage in cases of such complications. However, the rates of postoperative morbidity and mortality have decreased due to advances in surgical techniques and perioperative care. Therefore, the disadvantages of prophylactic drains have been questioned of late.

A previous investigation revealed that prophylactic drains do not reduce complications following hepatectomy, colorectal resection, and appendectomy [4]. Moreover, drains were demonstrated to be even harmful after hepatectomy in chronic liver disease and appendectomy [4]. Regarding gastrectomy, few studies have investigated prophylactic drainage [4–7]. However, data regarding prophylactic drainage after laparoscopic gastrectomy are minimal [1, 8].

Therefore, this study aimed to evaluate the effect of prophylactic drains after laparoscopic gastrectomy for treating gastric cancer.

## Methods

### Participants

We reviewed patients who received gastrectomy for histologically confirmed gastric cancer in our institution between April 2011 and March 2017. Those who underwent open surgery or proximal gastrectomy with lower esophagectomy for esophagogastric junction (EGJ) cancer were excluded. In addition, patients who received simultaneous complicated surgery for other organ diseases were excluded. The outcomes of patients who received laparoscopic gastrectomy with and without prophylactic drainage were compared. Propensity score matching was used to minimize potential selection bias.

### Operative indication and procedure

All radical surgeries for treating gastric cancer were performed laparoscopically irrespective of the clinical stage, unless an emergent situation such as perforation or acute bleeding existed.

The extent of gastrectomy and lymph node dissection was determined based on the Japanese gastric cancer treatment guidelines [9, 10]. A splenectomy was performed when the tumor was found to be located on the greater curvature of the upper third of the stomach. Moreover, combined resection of other organs such as distal pancreatectomy, transverse colectomy, and partial hepatectomy was performed when cancer involvement was suspected and when R0 resection was possible.

All reconstruction procedures were intracorporeally performed. Billroth I using delta-shaped anastomosis [11] and Roux-en-Y using a functional end-to-end method [12, 13] were the preferred choices of reconstruction procedures after distal and total gastrectomies, respectively.

The decision to use a prophylactic drain was made by the surgeon. After January 2015, drains were not used in principle owing to the low incidence of postoperative complications. However, in case of drain requirement, a closed passive drain was used.

Operations were performed by or under the guidance of qualified surgeons registered in the Japanese Society for Endoscopic Surgery [14].

### Surgical outcome assessment

Surgical outcomes in this study included operative mortality, the incidence of postoperative complications, the number of days after the surgery until the initiation of a soft diet, and the length of postoperative hospital stay. Postoperative complications included any adverse events determined as grade II or more using the Clavien–Dindo classification within 30 days after the surgery. Operative mortality was defined as death occurring within 30 days after the surgery.

### Statistical analysis

All statistical analyses were performed using R version 3.3.2 (the R foundation for statistical computing, Vienna, Austria). Intergroup comparisons were performed using Student’s *t* test for continuous variables and two-tailed *χ*^2^ test for discrete variables. Statistical significance was set a priori at *P* < 0.05.

Propensity score was calculated using a multiple logistic regression model for the variables shown in Table 1. Propensity score matching was then conducted by nearest-neighbor matching without replacement with an algorithm of 1:1 matching. A caliper width of 0.25 of the standard deviation of the logit of the propensity score was used.

**Table 1:** Demographic and perioperative characteristics before and after propensity score matching

	Before propensity score matching			After propensity score matching		
	Drain	No drain	*P* value	Drain	No drain	*P* value
	(*n* = 327)	(*n* = 301)		(*n* = 145)	(*n* = 145)	
Sex, *n* (%)			0.048			0.613
Male	237 (72.5)	196 (65.1)		102 (70.3)	97 (66.9)	
Female	90 (27.5)	105 (34.9)		43 (29.7)	48 (33.1)	
Age, mean ± SD, years	68.8 ± 10.0	69.2 ± 11.3	0.629	68.3 ± 10.7	68.4 ± 11.6	0.937
Body mass index (BMI), mean ± SD	22.7 ± 3.4	22.6 ± 3.0	0.527	22.7 ± 3.1	22.6 ± 2.9	0.895
Performance status (ECOG), *n* (%)			0.284			0.966
0	193 (59.0)	158 (52.5)		88 (60.7)	88 (60.7)	
1	112 (34.3)	114 (37.9)		46 (31.7)	44 (30.3)	
2	21 (6.4)	26 (8.6)		11 (7.6)	12 (8.3)	
3	1 (0.3)	3 (1.0)		0 (0)	1 (0.7)	
Comorbidity, *n* (%)						
Ischemic heart disease	27 (8.3)	17 (5.6)	0.214	8 (5.5)	10 (6.9)	0.809
Heart failure	2 (0.6)	4 (1.3)	0.434	1 (0.7)	1 (0.7)	1
Hypertension	127 (38.8)	120 (39.9)	0.807	57 (39.3)	60 (41.4)	0.811
Diabetes mellitus	51 (15.6)	43 (14.3)	0.656	22 (15.2)	21 (14.5)	1
Chronic hepatitis/cirrhosis	20 (6.1)	6 (2.0)	0.015	3 (2.1)	5 (3.4)	0.723
Hemodialysis	2 (0.6)	2 (0.7)	1	0 (0)	1 (0.7)	1
Ventilatory impairment	69 (21.1)	58 (19.3)	0.619	29 (20.0)	25 (17.2)	0.651
Regular steroid use	9 (2.8)	3 (1.0)	0.146	4 (2.8)	3 (2.1)	1
Anti-thrombotic therapy	47 (14.4)	34 (11.3)	0.284	17 (11.7)	21 (14.5)	0.602
History of abdominal surgery	122 (37.3)	105 (34.9)	0.561	57 (39.3)	51 (35.2)	0.544
Depth of invasion, *n* (%)			< 0.001			0.943
cT1	125 (38.2)	180 (59.8)		80 (55.2)	75 (51.7)	
cT2	60 (18.3)	53 (17.6)		24 (16.6)	26 (17.9)	
cT3	62 (19.0)	33 (11.0)		19 (13.1)	19 (13.1)	
cT4	80 (24.5)	35 (11.6)		22 (15.2)	25 (17.2)	
Node metastasis, *n* (%)			< 0.001			0.48
cN0	230 (70.3)	253 (84.1)		118 (81.4)	112 (77.2)	
cN1	48 (14.7)	24 (8.0)		10 (6.9)	17 (11.7)	
cN2	39 (11.9)	20 (6.6)		12 (8.3)	13 (9.0)	
cN3	10 (3.1)	4 (1.3)		5 (3.4)	3 (2.1)	
Preoperative chemotherapy, *n* (%)	26 (8.0)	13 (4.3)	0.069	9 (6.2)	9 (6.2)	1
Operative procedure, *n* (%)			< 0.001			
Distal gastrectomy (DG)	173 (52.9)	256 (85.0)		104 (71.7)	109 (75.2)	
Total gastrectomy (TG)	134 (41.0)	40 (13.3)		34 (23.4)	32 (22.1)	
Remnant gastrectomy	20 (6.1)	5 (1.7)		7 (4.8)	4 (2.8)	
Extent of lymphadenectomy, *n* (%)			< 0.001			0.878
DG D1+ or less	86 (26.3)	167 (55.5)		58 (40.0)	63 (43.4)	
DG D2	87 (26.6)	89 (29.6)		46 (31.7)	46 (31.7)	
TG D1+ or less	115 (35.2)	43 (14.3)		38 (26.2)	34 (23.4)	
TG D2	39 (11.9)	2 (0.7)		3 (2.1)	2 (1.4)	
Combined resection of other organs, *n* (%)	40 (12.2)	4 (1.3)	< 0.001	3 (2.1)	2 (1.4)	1
Type of reconstruction, *n* (%)			< 0.001			0.923
Billroth I	108 (33.0)	189 (62.8)		74 (51.0)	80 (55.2)	
Billroth II	44 (13.5)	45 (15.0)		19 (13.1)	20 (13.8)	
Roux-en-Y (DG)	22 (6.7)	22 (7.3)		11 (7.6)	9 (6.2)	
Roux-en-Y (TG; functional end-to-end)	101 (30.9)	40 (13.3)		34 (23.4)	31 (21.4)	
Roux-en-Y (TG; overlap)	51 (15.6)	5 (1.7)		7 (4.8)	5 (3.4)	
Roux-en-Y (TG; circular stapler)	1 (0.3)	0 (0)		0 (0)	0 (0)	
R status, *n* (%)			0.004			0.195
R0	286 (87.5)	285 (94.7)		139 (95.9)	132 (91.0)	
R1	24 (7.3)	7 (2.3)		2 (1.4)	7 (4.8)	
R2	17 (5.2)	9 (3.0)		4 (2.8)	6 (4.1)	
Blood loss, mean ± SD, ml	52.7 ± 138.3	18.7 ± 62.0	< 0.001	22.5 ± 72.1	26.8 ± 80.8	0.634
Operation time, mean ± SD, min	349 ± 78	268 ± 62	< 0.001	308 ± 57	304 ± 62	0.598

## Results

A total of 779 patients who underwent surgery for gastric cancer were reviewed. Of these, 45 patients who underwent proximal gastrectomy for EGJ cancer, 83 who underwent open gastrectomy (including four cases converted from laparoscopic surgery), and 21 who underwent a simultaneous surgery for other diseases such as colorectal cancer (*n* = 14), hepatic cancer (*n* = 1), familial adenomatous polyposis (*n* = 1), choledocholithiasis (*n* = 1), breast cancer (*n* = 2), ovarian tumor (*n* = 1), and malignant lymphoma of the cecum (*n* = 1) were excluded. One patient who underwent emergent laparoscopic surgery for a gastric cancer perforation and one patient with insufficient clinical data on our database were also excluded.

Thus, 628 patients who received elective laparoscopic gastrectomy were enrolled in this study. In this cohort, prophylactic drain was used for 327 patients (52.1%). Table 1 shows the perioperative characteristics of the patients with (drain group) and without a drain (no-drain group). Significant differences were noted between the two groups with respect to sex, chronic liver disease, clinical T and N stages, the extent of gastrectomy and lymphadenectomy, combined resection of other organs, the type of reconstruction, the amount of blood loss, the length of the operation time, and R status. Table 2 shows the outcomes among patients with each characteristic in whole enrolled population.

**Table 2:** Outcomes among patients with each characteristic before propensity score matching

	Postoperative complication ≥grade II*		Postoperative complication ≥grade III*		Initiation of soft diet (days)		Postoperative hospital stay (days)	
	Drain	No drain	Drain	No drain	Drain	No drain	Drain	No drain
	*n* (%)	*n* (%)	*n* (%)	*n* (%)	Median (range)	Median (range)	Median (range)	Median (range)
Depth of invasion								
cT1 (*n* = 125 vs 180)	33 (26.4)	22 (12.2)	9 (7.2)	2 (1.1)	5 (3–47)	4 (3–33)	13 (7–82)	11 (3–65)
cT2 (*n* = 60 vs 53)	15 (25.0)	7 (13.2)	7 (11.7)	1 (1.9)	5 (3–44)	4 (3–45)	11.5 (8–104)	12 (7–57)
cT3 (*n* = 62 vs 33)	12 (19.4)	4 (12.1)	3 (4.8)	0 (0)	5 (3–29)	5 (3–18)	13 (8–92)	13 (6–56)
cT4 (*n* = 80 vs 35)	18 (22.5)	5 (14.3)	3 (3.8)	1 (2.9)	5 (3–81)	4 (3–67)	14 (9–114)	11 (4–90)
Node metastasis								
cN0 (*n* = 230 vs 253)	59 (25.7)	32 (12.6)	16 (7.0)	3 (1.2)	5 (3–54)	4 (3–45)	13 (7–82)	12 (3–65)
cN1 (*n* = 48 vs 24)	12 (25.0)	3 (12.5)	5 (10.4)	0 (0)	5 (3–30)	4 (3–8)	13 (10–104)	11.5 (6–30)
cN2 (*n* = 39 vs 20)	6 (15.4)	2 (10.0)	0 (0)	1 (5.0)	5 (3–31)	5 (3–67)	12 (9–60)	11.5 (4–90)
cN3 (*n* = 10 vs 4)	1 (10.0)	1 (25.0)	1 (10.0)	0 (0)	4.5 (3–81)	3.5 (3–5)	13.5 (10–114)	11.5 (10–27)
Operative procedure								
Distal gastrectomy (DG) (*n* = 173 vs 256)	34 (19.7)	33 (12.9)	8 (4.6)	4 (1.6)	4 (3–54)	4 (3–67)	12 (7–59)	11 (4–90)
Total gastrectomy (TG) (*n* = 134 vs 40)	36 (26.9)	5 (12.5)	12 (9.0)	0 (0)	5 (3–81)	5 (3–18)	12 (8–114)	12 (4–56)
Remnant gastrectomy (*n* = 20 vs 5)	8 (40.0)	0 (0)	2 (10.0)	0 (0)	6.5 (4–22)	5 (3–5)	16.5 (9–82)	11 (3–12)
Extent of lymphadenectomy								
DG D1+ or less (*n* = 86 vs 167)	19 (22.1)	24 (14.4)	5 (5.8)	4 (2.4)	5 (3–21)	4 (3–67)	12 (9–52)	11 (4–90)
DG D2 (*n* = 87 vs 89)	15 (17.2)	9 (10.1)	3 (3.4)	0 (0)	4 (3–54)	4 (3–12)	11 (7–59)	12 (6–34)
TG D1+ or less (*n* = 115 vs 43)	31 (27.0)	4 (9.3)	11 (9.6)	0 (0)	5 (3–44)	5 (3–18)	13 (8–92)	12 (3–56)
TG D2 (*n* = 39 vs 2)	13 (33.3)	1 (50.0)	3 (7.7)	0 (0)	6 (3–81)	6 (5–7)	14 (9–114)	14 (14–14)
R status								
R0 (*n* = 286 vs 285)	67 (23.4)	36 (12.6)	18 (6.3)	3 (1.1)	5 (3–47)	4 (3–45)	13 (7–92)	12 (3–65)
R1 (*n* = 24 vs 7)	5 (20.8)	0 (0)	2 (8.3)	0 (0)	5.5 (3–54)	4 (3–6)	13.5 (9–104)	13 (10–34)
R2 (*n* = 17 vs 9)	6 (35.3)	2 (22.2)	2 (11.8)	1 (11.1)	6 (4–81)	4 (3–67)	16 (10–114)	11 (4–90)
Blood loss								
< 500 ml (*n* = 322 vs 300)	77 (23.9)	38 (12.7)	22 (6.8)	4 (1.3)	5 (3–81)	4 (3–67)	13 (7–114)	11.5 (3–90)
≥ 500 ml (*n* = 5 vs 1)	1 (20.0)	0 (0)	0 (0)	0 (0)	6 (4–21)	7	13 (11–31)	13

*Clavien–Dindo classification

**Fig. 1:**
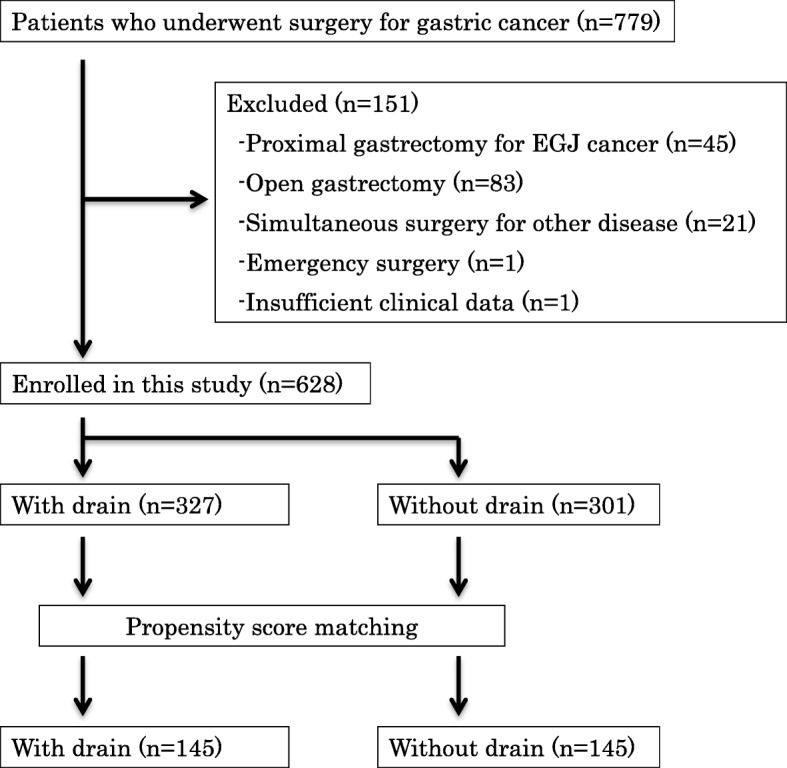
CONSORT diagram. Flowchart of the patients assessed in this study

After propensity score matching, data of 145 pairs of patients were extracted. The flow chart of patients assessed in this study is shown in Fig. 1. Perioperative characteristics after propensity score matching are additionally indicated in Table 1. No significant differences were noted in perioperative characteristics between the drain and no-drain groups after propensity score matching.

Table 3 shows the primary outcomes of this study. No significant differences were noted in the incidence of postoperative complications between the drain and no-drain groups (19.3% vs 11.0%, *P* = 0.071). The number of days after surgery until the initiation of soft diet (6.3 ± 7.4 vs 4.9 ± 2.9 days, *P* = 0.036) and the length of postoperative hospital stay (15.7 ± 12.9 vs 13.0 ± 6.3 days, *P* = 0.023) were greater in the drain group than in the no-drain group. No hospital deaths were observed within 30 days after surgery; three and one patients in the drain and no-drain groups, respectively, had complications that required reoperation within 30 days after the surgery. The breakdown of postoperative complications is shown in Table 4.

**Table 3:** Comparison of surgical outcomes between the drain and no drain groups in the matched cohort

	Drain (*n* = 145)	No drain (*n* = 145)	*P* value
Postoperative complications ≥grade II*, *n* (%)	28 (19.3)	16 (11.0)	0.071
Postoperative complications ≥grade III*, *n* (%)	8 (5.5)	3 (2.1)	0.218
Initiation of soft diet, mean ± SD, days	6.3 ± 7.4	4.9 ± 2.9	0.036
Postoperative hospital stay, mean ± SD, days	15.7 ± 12.9	13.0 ± 6.3	0.023

*Clavien–Dindo classification

**Table 4:** Postoperative complications in the drain and no drain groups in the matched cohort

	Drain (*n* = 145)	No drain (*n* = 145)
Grade II*		
Anastomotic leakage	2	1
Pancreatic fistula	6	1
Intra-abdominal abscess	2	3
Intra-abdominal bleeding	1	0
Ileus	1	0
Chylous ascites	1	1
Pneumonia	2	2
Delayed gastric emptying	0	1
Liver injury	2	1
Fever of unknown origin	1	2
Pseudomembranous colitis	0	1
Wound infection	1	0
Catheter-related infection	1	0
Grade III*		
Anastomotic leakage	3	0
Pancreatic fistula	2	1
Ileus	1	1
Pleural effusion	0	1
Anastomotic stenosis	1	0
Hepatorenal insufficiency	1	0
30-day mortality	0	0
Reoperation	3	1

*Clavien–Dindo classification

## Discussion

The results of the present study do not support the routine use of prophylactic drains following laparoscopic gastrectomy. Prophylactic drains are typically used for the early detection and prevention of the aggravation of postoperative complications. The present study, however, revealed no significant differences between the incidence and the severity of postoperative complications. Rather, complications tended to occur in the drain group. Moreover, in the drain group, the number of days after surgery until the initiation of soft diet and postoperative hospital stay were greater than those in the no-drain group, suggesting that drains do not additionally benefit or may even prove to be harmful for managing postoperative complications.

Few studies have been reported on the clinical value of prophylactic drains following surgeries for gastric cancer. Petrowsky et al. reported that the use of drains after total gastrectomy is justified because anastomotic leakage after total gastrectomy can cause life-threatening mediastinitis [4]; however, these conclusions were not based on comparative studies. We found three randomized controlled trials (RCTs) comparing drain use and no-drain use in patients who underwent gastrectomy [5–7]; however, none of the trials supported the use of prophylactic drainage after a subtotal or total gastrectomy.

Similarly, data regarding the use of prophylactic drains after laparoscopic gastrectomy is limited [1, 8], and to the best of our knowledge, there are no RCTs evaluating this use of prophylactic drains. Since Kitano et al. reported the first laparoscopy-assisted gastrectomy in 1994 [15], the number of patients that are being treated using the laparoscopic technique is increasing [16]. The safety associated with the use of laparoscopic gastrectomy is of great concern; however, advances in operative techniques and laparoscopic instruments have led to the standardization of laparoscopic gastrectomy, and its overall safety is gradually being clarified [17–19]. Considering the low incidence of postoperative complications (5.1–11.6%), the necessity of prophylactic drains should be evaluated.

In the present study, we included almost all patients with gastric cancer who were treated using laparoscopic gastrectomy, regardless of their comorbidity, the clinical stage of cancer, and the extent of the surgery. This comprehensive inclusion is more likely to reflect actual clinical settings. To minimize the risk of confounding variables, propensity score matching was used. Although RCTs have been considered the gold standard for therapeutic evaluation, assessing surgical procedures in RCTs raises several methodological and practical challenges for surgical research [20]. Recently, a study comparing the effects of treatment estimated from observational studies using propensity score analysis and those from RCTs performed for the same clinical question in the surgical field revealed no statistically significant differences [21]. However, this study contains a limitation due to historical nature of the study design. Since we changed the indication of prophylactic drains during the study period, drains were more likely to be placed to the patients who received surgery in the early period. Therefore, potential improvement in surgical technique and perioperative care during the study period may affect the outcomes. Even considering such a historical effect, omitting prophylactic drains was safe without increased morbidity.

## Conclusion

In conclusion, this study suggests that the routine use of prophylactic drainage after laparoscopic gastrectomy for gastric cancer is not always necessary, although caution should be exercised when making clinical decisions owing to the retrospective design of this study.

## Data Availability

The dataset used and analyzed during the current study is available from the corresponding author on reasonable request.

## Abbreviations

EGJ: Esophagogastric junction
RCTs: Randomized controlled trials
